# Too Aggressive Drop in Blood Pressure in a Hypertensive Male Leading to “Man-in-the-Barrel Syndrome”

**DOI:** 10.1155/2020/8855574

**Published:** 2020-09-24

**Authors:** Chamara Dalugama, Achila Jayasinghe, Udaya Ralapanawa, Shamali Abeygunawardena, Thilak Jayalath

**Affiliations:** ^1^Department of Medicine, University of Peradeniya, Peradeniya, Sri Lanka; ^2^Teaching Hospital, Peradeniya, Sri Lanka

## Abstract

**Introduction:**

“Man-in-the-barrel syndrome” is a neurological phenotype with brachial diplegia, normal sensation, and preserved motor function of the lower limb. It has been described in various neuropathological conditions affecting the cerebral hemispheres, pons, upper spinal cord, and peripheral neurons. Severe hypotension leading to watershed infarctions leading to this phenotype has been reported. We describe the first case of “man-in-the-barrel syndrome” in a patient with a precipitous drop in blood pressure following oral antihypertensive medications. *Case Presentation*. A 75-year-old Sri Lankan male presented following a generalized tonic-clonic seizure to a tertiary care hospital. Upon recovery, he was noted to have severe brachia diplegia affecting shoulder movements with preserved hand muscle power and motor functions of the lower limb. The previous day, he was newly diagnosed with markedly elevated blood pressure without acute end organ involvement. Treatment with three antihypertensives had been initiated. Noncontrast CT of the brain revealed watershed infarctions affecting both cerebral hemispheres.

**Conclusion:**

It is generally unwise to lower blood pressure very rapidly, as ischemic damage can occur in vascular beds that are habituated to high levels of blood pressure in the brain. Ischemic damage caused by rapid lowering of blood pressure may rarely result in “man-in-the-barrel syndrome” leading to severe functional disability.

## 1. Introduction

Undiagnosed hypertension is a killer in disguise which can surface as hypertensive emergencies or urgencies and may cause hypertension-mediated end organ involvement [1–3]. Prompt but careful reduction of blood pressure is needed in a hypertensive emergency, whereas in markedly elevated blood pressure with end organ involvement, blood pressure needs to be brought down slowly over days to prevent cerebral, cardiac, or renal ischemia [4–6].

“Man-in-the-barrel syndrome” is a rare phenotype with severe brachial diplegia and intact motor functions of the lower limb, where the arms of the man appear to be confined to a “barrel” as the name implies [7, 8]. Multitudes of etiologies have been described to cause this phenotype, including severe and acute cerebral hypoperfusion leading to bilateral watershed infarctions [9].

We describe the first case where oral antihypertensive medications causing precipitous drop in blood pressure were leading to the “man-in-the-barrel” phenotype.

## 2. Case Presentation

We report a case of a 75-year-old Sri Lankan male who presented to a general practitioner for routine health checkup. A high blood pressure of 220/120 mmHg was found. His past medical record was unremarkable. At the time of presentation to the general practitioner, he did not have any symptoms or signs of new end organ involvement in the form of heart failure, aortic dissection, renal involvement, hypertensive encephalopathy, or retinal involvement. Treatment was initiated with losartan potassium 50 mg twice a day, hydrochlorothiazide 25 mg morning, and prazocin 1 mg three times daily at the same time by the general practitioner.

The next day morning, the patient was admitted to the Accident and Emergency department following a generalized tonic-clonic seizure. On admission, his blood pressure had been 80/50 mmHg. His blood sugar was 120 mg/dL. Urgent electrolyte profile revealed normal serum sodium, potassium, calcium, and magnesium. He was treated with intravenous fluids and the blood pressure improved.

After regaining consciousness after the seizure, the patient complained about difficulty in raising the arms above the head. Family members who witnessed the event assured that he did not have any trauma to the shoulder girdle during the seizure. His distal arm and finger movements were well preserved. He could walk without support and could get up on his own from a squatting position.

On neurological examination, we found a man who was fully conscious, rational, and oriented. His cranial nerve examination was normal with normal bilateral fundi.

Upper limb examination revealed reduced muscle power proximally with bilateral shoulder abduction 3/5, shoulder extension 4/5, and shoulder flexion 4/5. Shoulder adduction was in full strength. His power at the elbow flexion and extension was normal and distal hand motor functions were normal. Tone in the proximal muscle group was reduced. Reflexes of the upper limbs were normal with normal sensory examination. Lower limb examination was neurologically normal with bilateral flexor plantar response (Videos 1 and 2).

**Figure 1:**
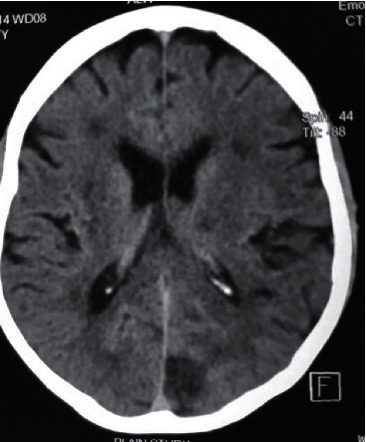
Noncontrast CT scan of the brain showing bilateral watershed infarctions in the fronto-parietal regions and a right-sided occipital infarction.

**Figure 2:**
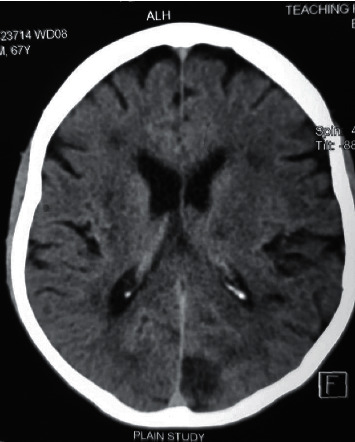
Noncontrast CT scan of the brain showing bilateral watershed infarctions in the fronto-parietal regions and a right-sided occipital infarction.

Noncontrast CT of the brain performed 24 hours after symptom onset revealed new bilateral hypodense areas involving parietal regions, suggestive of watershed infarctions and an occipital infarct (Figures 1 and 2) in comparison to the CT performed on admission. Shoulder joint X-rays excluded fractures or dislocations in the shoulder girdle. X-ray of the cervical spine did not show any evidence of cervical spondylosis. Electromyogram and nerve conduction studies excluded a myopathic disorder or cervical root pathology. Carotid artery duplex sonography was performed in bilateral carotid arteries and excluded carotid artery stenosis. 2D echocardiogram showed left ventricular hypertrophy without evidence of intracardiac thrombi or valvular dysfunction. Patient's resting ECG with a long rhythm strip was showing left ventricular strain without evidence of any arrhythmia and 24-hour holter monitoring did not show any clinically significant rhythm abnormality.

Biochemical blood measurements revealed hemoglobin of 14.6 g/dL with a platelet count of 340 *∗* 10^6^/L. Serum creatinine was 90 mmol/L with blood urea of 6.5 mmol/L. Electrolyte profile revealed sodium of 138 mmol/L and potassium of 4.5 mmol/L. His coagulation profile was normal. EEG did not show evidence of seizure activity.

He was started on antiplatelets and statin. Physiotherapy and occupational therapy were arranged and the patient was started on phenytoin sodium for seizures. The blood pressure was monitored and controlled very cautiously with low doses of losartan potassium. He made a slow recovery with mild residual weakness in the shoulder abduction.

## 3. Discussion

Hypertension is a major killer in the world and is responsible for nearly nine million deaths a year according to the WHO [1]. Hypertension is defined as office systolic blood pressure values ≥140 mmHg and/or diastolic BP (DBP) values ≥90 mmHg by the European Society of Cardiology [2]. Hypertension emergency is defined as severe hypertension (grade 3) which is associated with acute hypertension-mediated organ damage. It may be life-threatening and immediate but careful reduction of blood pressure with intravenous antihypertensives is needed [3]. Severe hypertension (grade 3) without end organ involvement is defined as a hypertensive urgency where blood pressure needs to be reduced slowly over a period of 24–48 hours using oral medications [4]. Excessive reduction in blood pressure in a patient with markedly elevated blood pressure without target organ damage is not recommended as it can precipitate renal, cerebral, or coronary ischemia [5, 6]. In this case, our patient had markedly elevated blood pressure without evidence of end organ involvement and unfortunately his blood pressure was lowered over few hours using 3 potent antihypertensive medications.

Cerebral autoregulation is the ability to maintain stable perfusion to the brain tissue to meet the metabolic demands of the brain despite changes in blood pressure [7]. Myogenic, neurogenic, endothelial, and metabolic responses have been described in this autoregulatory process [8]. In longstanding untreated hypertension as in this case, it is well known that the static autoregulatory curve shifts rightward, placing patients at risk of impaired ability to tolerate hypotension [9]. Thus, too aggressive lowering of long standing blood pressure could have undesirable effects on brain function.

“Man-in-the-barrel syndrome” is a rare but well-reported syndrome of brachial diplegia. It was first described by Dide in 1917 [10]. Different terms were coined in medical literature to describe the same clinical presentation over the years such ascruciate paralysis [11], distal field infarction [12], and brachial amyotrophic diplegia syndrome [13]. The classic clinical presentation is paralysis of the upper extremity, more pronounced proximally than distally, with intact motor functions of the lower limbs. As the subject is unable to move his arms, it appears that the upper limbs are confined in a barrel [14]. In our case, the patient had marked weakness in the proximal upper limb muscles, but motor functions of the distal hand muscles and lower limb muscles were well preserved.

The phenotype of “man-in-the-barrel syndrome” has been described in many neuropathological conditions localizing to the cerebral cortex, pons, anterior horn cells of the cervical spinal cord, and peripheral nerves [15–18]. Acute systemic hypotension is the most commonly described aetiology leading to this syndrome. Several such cases have been described following cardiorespiratory arrest and postoperative hypotension [19–21]. We describe the first case of precipitous iatrogenic lowering of blood pressure using multiple antihypertensive drugs leading to this phenotype. Rapid lowering of blood pressure can result in ischemic lesions in the border zone between the middle and anterior cerebral artery territories. This is the location of the precentral gyrus, which is the motor region responsible for motor activity of the arm and the shoulder [22]. In our patient, CT scans of the brain revealed bilateral hypodense areas involving watershed zones. This suggested the development of ischemic lesion following rapid drop in blood pressure. His bilateral carotid arteries were normal indicating acute severe blood pressure drop is the sole cause for the bilateral watershed infarctions.

Many other etiologies have been described leading to a phenotypic presentation of “man-in-the-barrel syndrome.” Multiple strokes [23] and metastases [24] involving bilateral cerebral cortices have been described with the syndrome. Telfer et al. described a case due to central pontine myelinolysis caused by hyponatremia [25]. Upper cervical cord lesions such as infarction or tumor have been found to cause similar phenotypes [26]. Amyotrophic lateral sclerosis (ALS) has also been described as a rare cause of “man-in-the-barrel syndrome” [13].

The prognosis of this condition is highly variable and it depends on the aetiology. Some case series with motor neuron disease reported mortality up to 90%. Other cases reported good recovery with minimum residual neurological deficits. Our patient's prognosis was good and his proximal weakness improved partially with physiotherapy.

## 4. Conclusion

In the absence of hypertension-mediated acute end organ damage, aggressive treatment of markedly elevated blood pressure should be avoided, as it can lead to grave consequences such as cerebral ischemia. “Man-in-the-barrel syndrome” is a rare phenotype that can be caused by multiple neuropathological processes localizing to the bilateral cerebral cortices, pons, upper spinal cord, or peripheral neurons. It can also be caused by watershed infarctions following cerebral ischemia, which may lead to severe functional impairment with a variable prognosis.

## Data Availability

No datasets were generated or analyzed during the current study.

## Supplementary Materials

Supplementary Materials Two MP4 video clips. (1) Video 1: the patient demonstrating bilateral brachial diplegia when asked to lift up the arms above the head. (2) Video 2: the patient demonstrating the intact motor functions of the lower limbs when asked to take a few steps on the ground.
